# Vitamin K Antagonists (VKAs) and Novel Oral Anticoagulants (NOACs) Safety Comparison Based on Data from EudraVigilance Database

**DOI:** 10.3390/hematolrep17050054

**Published:** 2025-10-18

**Authors:** Pier Paolo Olimpieri, Fanny Erika Palumbo, Gaetano Giuffrida, Edoardo Milanetti, Cecilia Gozzo, Elisa Lucia Scebba, Giovanni Luca Romano, Giovanni Enrico Lombardo, Andrea Duminuco, Calogero Vetro, Davide Giuseppe Castiglione, Giuseppe Alberto Palumbo, Salvatore Scarso, Filippo Drago, Lucia Gozzo

**Affiliations:** 1 Italian Medicines Agency, 00187 Rome, Italy; p.olimpieri@aifa.gov.it; 2 UOC Ematologia con Trapianto di Midollo, A.O.U. Policlinico “G. Rodolico”-S. Marco, 95123 Catania, Italy; gaegiuffrida@gmail.com (G.G.); andrea.duminuco@gmail.com (A.D.); palumbo.ga@gmail.com (G.A.P.); 3 Department of Physics, Sapienza University, Piazzale Aldo Moro 5, 00184 Rome, Italy; edoardo.milanetti@uniroma1.it; 4 Radiology Unit, Humanitas Istituto Clinico Catanese, 95045 Catania, Italy; ceciliagozzo91@gmail.com; 5 Department of Clinical and Experimental Medicine, University of Messina, 98125 Messina, Italy; elisascebba@tiscali.it; 6 Department of Medicine and Surgery, University of Enna “Kore”, 94100 Enna, Italy; giovanniluca.romano@unikore.it (G.L.R.); giovannienrico.lombardo@unikore.it (G.E.L.); 7 Hematology and Bone Marrow Transplantation Unit, Hospital of Bolzano (SABES-Azienda Sanitaria dell’ Alto Adige), Teaching Hospital of Paracelsus Medical University, 39100 Bolzano, Italy; gerovetro@gmail.com; 8 Radiology I Unit, University Hospital Policlinico “G. Rodolico”-S. Marco, 95123 Catania, Italy; davidegiuseppecastiglione@gmail.com; 9 Department of Medical Surgical Sciences and Advanced Technologies “GF Ingrassia”, University Hospital Policlinico “G. Rodolico”-S. Marco, 95123 Catania, Italy; 10 Department of Infectious, Tropical Diseases and Microbiology, IRCCS Sacro Cuore Don Calabria Hospital, 37024 Negrar di Valpolicella, Italy; salvatorescarso31@gmail.com; 11 Department of Biomedical and Biotechnological Sciences, University of Catania, 95123 Catania, Italy; f.drago@unict.it; 12 Centre for Research and Consultancy in HTA and Drug Regulatory Affairs (CERD), University of Catania, 95123 Catania, Italy; 13 Clinical Trial Unit, Azienda Ospedaliero Universitaria Policlinico “G. Rodolico–S. Marco”, 95123 Catania, Italy; luciagozzo86@icloud.com

**Keywords:** novel oral anticoagulants, Vitamin K Antagonists, adverse events, EudraVigilance, safety

## Abstract

**Background**: Clinical trials comparing novel oral anticoagulants (NOACs) with warfarin reported a lower mortality rate and a reduced incidence of bleeding with NOACs. However, these studies do not allow for final conclusions about safety. Moreover, direct comparisons among NOACs are not available. **Objectives**: The aim of this study was to analyze data from EudraVigilance in order to compare OAC safety profiles. **Methods**: We searched for all suspected adverse drug reactions (ADRs) from OACs collected in the EudraVigilance up to March 2019. We calculated the reporting odds ratios (RORs) in order to assess the risk of reporting specific ADRs among drugs. Moreover, OAC safety profiles were investigated through correspondence analysis and visualized in contribution biplots. **Results**: A total of 244,149 individual case safety reports (ICSRs; 431,354 ADRs) related to OACs were retrieved from EudraVigilance. About 80% of ICSRs refer to NOACs, especially rivaroxaban. Gastrointestinal (Gastr) and central nervous system (Nerv) disorders were the most represented categories. More than 90% of ADRs were serious and almost 9% fatal, with the highest ROR reported for dabigatran. Both fatal and non-fatal ADRs reported for Vitamin K Antagonists (VKAs) differed from those reported for NOACs. Among the latter, dabigatran and rivaroxaban showed similar profiles, while apixaban differed from all other OACs, even in the case of fatal ADRs. **Conclusions**: As expected, data collected from EudraVigilance showed differences among drugs, probably related to their specific characteristics and/or the peculiar utilization in clinical practice. Further investigations are needed to better compare the safety profile of OACs.

## 1. Introduction

Anticoagulants represent the drugs of choice for the prevention and treatment of venous thromboembolism and to reduce the risk of stroke in patients with atrial fibrillation [1,2,3,4,5,6]. Novel oral anticoagulants (NOACs) have changed the landscape of treatment in these clinical conditions, demonstrating non-inferiority in efficacy and safety compared with warfarin in randomized controlled trials (RCTs) with fewer treatment-related issues [2,3,7,8,9]. The primary safety concern with all oral anticoagulants (OACs) is obviously the risk of bleeding, which is strictly related to the pharmacological effect [10,11,12,13,14]. Intracranial bleeding is the most feared adverse drug reaction (ADR), due to irreversible sequelae and a high rate of mortality, whereas gastrointestinal bleeding is more common but less often fatal. Differences in bleeding sites have been observed with NOACs in comparison to Vitamin K Antagonists (VKAs) [15].

More generally, beyond the differences in terms of the mechanisms of action, food effect, drug–drug interactions, and renal clearance, indirect comparisons and network meta-analyses demonstrated that NOACs have generally similar efficacy but different safety profiles [16,17,18,19].

However, until now, there have been no head-to-head RCTs comparing NOACs [20]. Even if available, clinical trials do not allow for final conclusions about safety, and are usually designed to evaluate efficacy above all, and include a small and selected well-monitored population, which do not reflect real-world conditions [21,22,23]. Therefore, the most commonly used approach for post-marketing surveillance still relies on spontaneous reporting systems: despite multiple limitations, such as underreporting and reporting biases, lack of information about drug exposure, and difficulty in distinguishing ADRs from other events, this approach remains the most efficient way to collect safety data [23,24].

This study aims to analyze data from a spontaneous reporting system in order to compare OAC safety profiles in real-world and long-term use.

## 2. Materials and Methods

We analyzed all suspected ADRs related to the use of the main OACs (warfarin, acenocumarol, dabigatran, rivaroxaban, apixaban, edoxaban) reported on EudraVigilance up to March 2019.

EudraVigilance is the European database maintained by the European Medicines Agency (EMA) since 2001 [25,26], which collects, manages and analyzes suspected ADRs to medicines authorized in the European Economic Area (EEA), submitted by European national regulatory authorities (or by marketing-authorization holders for ADRs occurring outside Europe). Each individual case safety report (ICSR) is composed of at least one report, which might be integrated with the follow-up. Non-serious ADRs occurring outside of the EEA are not included.

Aggregated data are freely accessible from the “European Database of Suspected Adverse Drug Reaction Reports” (http://www.adrreports.eu/, accessed on 14 October 2025). They are coded by the Medical Dictionary for Regulatory Activities (MedDRA), and classified by age classes, sex, system organ classes (SOCs), seriousness (serious, non-serious, not specified) and outcome (fatal, not recovered, not specified, recovered, recovered with sequelae, recovering, unknown) [27].

According to criteria from international guidelines, serious ADRs are those resulting in death, life-threatening events, requiring or prolonging hospitalization, resulting in persistent or significant disability/incapacity or congenital anomaly/birth defect, or any other medical event deemed to be important due to major clinical consequences [28].

The Reporting Odds Ratio (ROR) was calculated as a measure of disproportionality to compare the risk of reporting specific ADRs among drugs [29].

Moreover, we calculated the indexed residuals for each specific drug–ADR pair, defined as the difference between the observed and expected values. Positive and negative values indicated that the observed values were higher and lower than expected, respectively. Expected values were obtained from the relative frequencies of an individual ADR and all the ADRs for a given drug (i.e., multiplying all column totals with all row totals in Tables S1 and S2).

Similarities in the safety profiles of OACs were investigated through correspondence analysis (CA) [29] and visualized with a contribution biplot [30]. CA is a multivariate dimensionality reduction method that provides a means to summarize data and visualize them in a two-dimensional space. In this way, new coordinates are obtained for each observation (i.e., the OACs), and the closer the points are, the more similar their safety profiles are. Moreover, variables (i.e., the ADRs) that contribute the most to the overall variability in the original dataset can be shown in a two-dimensional plot (called a contribution biplot) as vectors, together with OAC coordinates, allowing an easier interpretation of which ADR is mainly accountable for the separation of safety profiles. Indeed, the longer the vector in the plot, the higher the contribution of the specific ADR to the definition of the safety profile representation [30].

We performed CA and generated the contribute biplots using R statistical software (R Core Team (2019). R: A language and environment for statistical computing. R Foundation for Statistical Computing Vienna, Austria. URL https://www.R-project.org/, accessed on 14 October 2025) and the libraries FactoMineR [31] and ggplot2 [32]. The minimum number of dimensions to analyze were selected according to Bendixen, Mike in 2003 [33], while the most important variables were identified using the average expected contribution calculated as a threshold, on the assumption of a uniform contribution for all the variables as the reciprocal of the number of variables (in other words: 1/number of considered ADRs).

In order to minimize the effect of possible confounders, we excluded the ADRs reported as ‘social circumstances’ (including conditions such as ‘elderly’, ‘family stress’, ‘economic problem’, ‘low income’) and fatal ADRs that are considered implausible or explainable by other conditions (such as ‘ear’ and ‘eye disorders’, ‘investigation’, ‘surgical procedures’, and ‘product issues’).

## 3. Results

**Figure 1:**
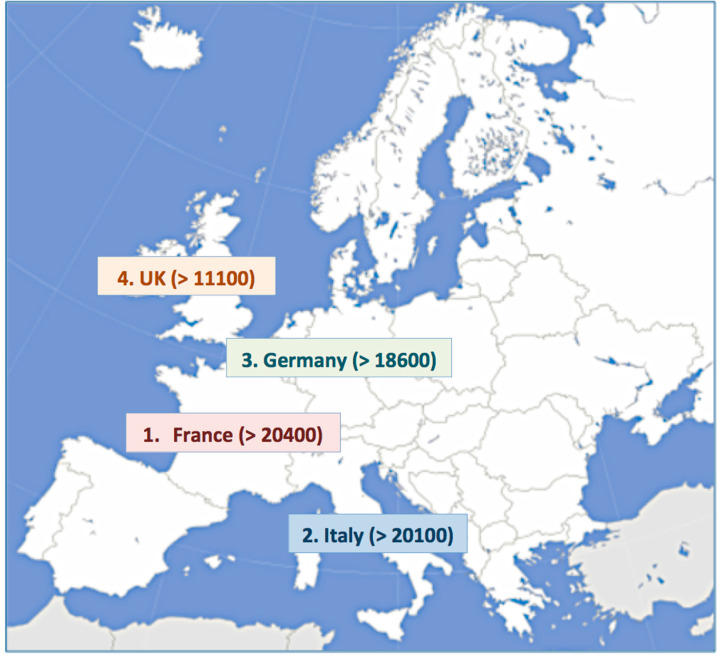
European countries with more than 10,000 ICSRs.

A total of 244,149 individual cases related to OACs were retrieved from the EudraVigilance database, corresponding to 431,354 ADRs (Table 1 and Table 2). About 80% of ICSRs refer to NOACs: in particular, rivaroxaban (41.6%). Comparing the number of ICRSs reported by European countries, we found that France and Italy represent the ones with the highest number of reports (more than 20,000; Figure 1), followed by Germany and the UK. In contrast with the other European nations, the drug with the highest rate of ADRs in Italy is warfarin (57.2%; Table 3), followed by dabigatran (15.8%).

Males and females are equally represented, with most of them belonging to the 65–85 age group. Moreover, a significant proportion of ICSRs have been reported for patients who were 85 and over (14.6%), in particular for edoxaban and acenocumarol (19.5% and 19.4%, respectively).

More than 90% of ADRs are serious, with rivaroxaban having the highest (95.5%; OR 1.95 IC 95 1.89–2.00) and edoxaban the lowest rate (70.9%; OR 0.15 IC 95 0.14–0.16). ADRs with a fatal outcome are overall 38,250 (8.9%), with the highest percentage reported for dabigatran (12.4%; OR 1.67 IC 95 1.62–1.71).

‘Gastrointestinal’ (‘Gastr’) and ‘nervous system’ (‘Nerv’) disorders are the most represented categories, accounting for 30.3% of ADRs and 39.0% of fatal ADRs (Supplementary Tables S1 and S2).

Table 4 and Table 5 show the percentages of indexed residuals for ADRs categorized according to SOCs. The larger positive differences between observed and expected ADRs have been found for warfarin and acenocumarol with ‘pregnancy, puerperium and perinatal conditions’ (‘Preg’; 255.4% and 149.8%, respectively) and ‘investigations’ (‘Inv’; 104.0% and 128.0%, respectively), for apixaban with ‘surgical and medical procedures’ (‘Surg’; 156.1%) and for edoxaban with ‘skin and subcutaneous tissue disorders’ (‘Skin’; 122.6%).

Dabigatran and rivaroxaban are the only two OACs where ‘Gastr’ (one of the most reported ADRs) has been found to be more likely (27.1% and 15.2%) than expected.

Large differences have been found associated between ‘Preg’ and warfarin (386.1%) and acenocumarol (72.1%), while also considering only fatal ADRs. In this case, the ‘Preg’ ADR is less likely observed with NOACs, with the only exception being edoxaban (208.8% more likely than expected). Fatal ‘general disorders and administration site conditions’ (‘Genrl’) have been found to be more likely for apixaban (95.3%) and less likely for all the other OACs. The same has been observed for ‘Gastr’ fatal ADR and dabigatran (53.9%). On the contrary, large positive differences between expected and observed values in rivaroxaban ADRs were not identified.

**Figure 2:**
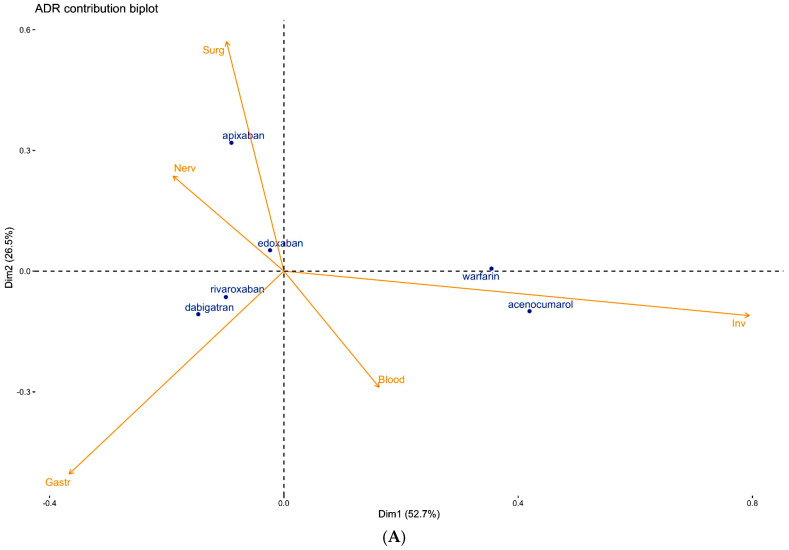

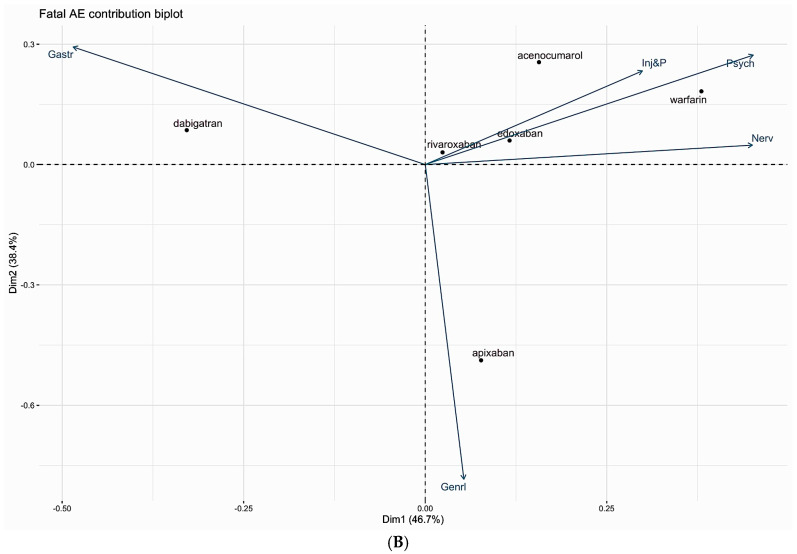
(**A**) Contribution biplot showing the determinant role of ADRs in separating the safety profiles of OACs. (**B**) Contribution biplot showing the determinant role of fatal ADRs in separating the safety profiles of OACs.

Using CA, we were able to separate ADRs in a space defined by only two dimensions (dm1 and dm2), accounting for about 80% of the whole dataset variance (79.2% and 85.1% for overall and fatal ADRs, respectively; see Figure 2A,B and Methods for further details). In particular, for overall ADRs, dm1 accounted for more than half (52.7%) and dm2 for about one third (26.5%) of the observed variance. 

In Figure 2A, warfarin and acenocumarol are well separated along the positive pole of dm1, rivaroxaban, and dabigatran in the negative quadrant of dm1 and dm2, apixaban far from all the other OACs in the further positive region of dm2, and edoxaban close to the origin.

The top 5 ADRs are represented by vectors, the length of which determines their contribution in the separation of OACs. ‘Inv’ is the main ADR responsible for the separation of VKAs from NOACs along dm1, while ‘Surg’ and ‘Gastr’ (dm2) show a determinant role in separating the apixaban safety profile from dabigatran and rivaroxaban. The exclusion of ‘Inv’ maintains the initial clustering (Supplementary Figure S1), although the two dimensions explain only about 60% of the overall variance. In this case, even edoxaban is separated from the origin dragged by ‘Nerv’.

The contribution biplot for fatal ADRs (Figure 2B) demonstrates again the separation along dm1 between VKAs and NOACs, with a greater distance between acenocumarol and warfarin. In this case, rivaroxaban and edoxaban, being close to the origin, have been playing a minor role in the analysis. Dabigatran and apixaban are further away in the negative poles of dm1 and dm2, respectively. The top fatal ADRs are ‘Genrl’, which is strongly associated with the negative pole of dm2, and therefore separating apixaban from the others OACs; ‘Gastr’, which equally contributes to both dimensions in the direction along which dabigatran drifted; ‘Nerv’; and ‘Psychiatric disorders’ (‘Psych’), which is involved in the separation of warfarin. The last relevant fatal ADRs identified by this analysis are ‘injury, poisoning and procedural complications’ (‘Inj&P’), which are partially associated with acenocumarol and warfarin.

**Figure 3:**
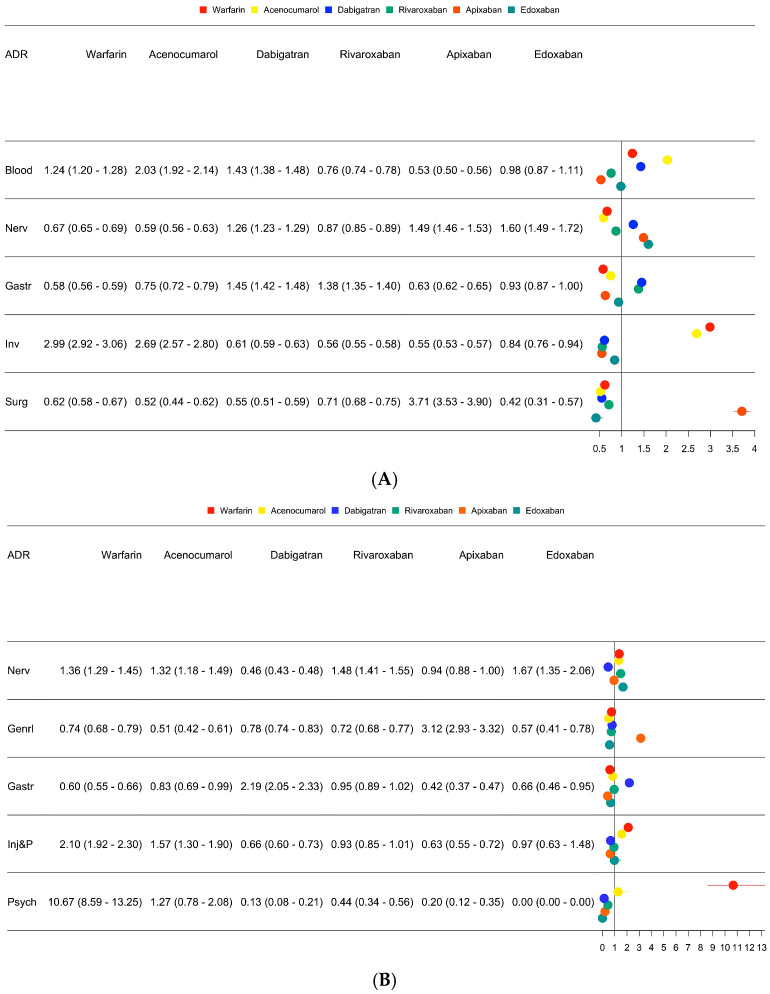
(**A**) ROR and their 95% confidence intervals for specific ADRs. ADRs reported into EudraVigilance for each drug have been compared with the related categories reported for the others. (**B**) ROR and their 95% confidence intervals for fatal ADRs. ADRs with a fatal outcome were 38,250 (8.9%) overall, and differences in the likelihood of reporting them were found among OACs.

Figure 3A,B show the ROR of the top 5 ADRs, which characterize the OAC safety profile according to our analysis (Supplementary Tables S3 and S4).

## 4. Discussion

OACs are included in the World Health Organization Model List of Essential Medicines [34], which represents a list of the most efficacious, safe and cost–effective medicines, selected on the basis of current and future public health relevance. Although it is still difficult to be treat some categories of patients with OAC [35], their use has increased over time and is expected to grow further, due to aging populations and the prevalence of chronic cardiovascular disease and AF risk factors [36]. Indeed, target patients are predominantly elderly, with high rates of cardiovascular risk factors and morbidity [37]. For more than 50 years since it was first approved for human use, warfarin was the only leading oral anticoagulant available [38]. NOACs have been approved by the European Union since 2008 [39], and the first authorization for the prevention of stroke and systemic embolism in patients with nonvalvular atrial fibrillation (NVAF) was granted for dabigatran in 2011. These drugs have overcome many of the practical limitations of VKAs, appearing to have at least similar efficacy, with fewer major bleeding events [20,40], while VKAs still mantain the main indication for splancnic thrombosis, deep vein thrombosis, antiphospholypid syndrome or prosthetic heart valve [41,42,43,44,45,46,47,48]. 

Several systematic reviews have concluded that the risk of major bleeding in NOACs is generally equivalent to or less than that with warfarin [49,50,51,52]. Therefore, they surpassed warfarin as the drug of choice, particularly for the management of AF [51], and a steady decrease in initiations of warfarin and an increase in NOACs were observed over time. This can, at least in part, explain the greater number of ADRs reported for NOACs on EudraVigilance compared to VKAs, despite their shorter post-marketing period and their better safety profile. However, we found differences in the rate of ADRs among the top European countries, with Italy being the only one with warfarin as the main drug according to the number of ADRs. As reported by the Italian Medicines Agency (AIFA) in the National Report on Medicines use in Italy [53], despite the progressive reduction in their use since the introduction of NOACs in 2013, in 2018 VKAs still represented the most prescribed OACs in Italy (DDD/1000 inhabitants/die = 4.6), followed by rivaroxaban (DDD/1000 inhabitants/die = 3.2), apixaban (DDD/1000 inhabitants/die = 3.0), dabigatran (DDD/1000 inhabitants/die = 2.2) and edoxaban (DDD/1000 inhabitants/die = 1.0) [54]. Comprehensive data about patients’ exposure in different countries are not available. The European and national guidelines did not favor any particular NOAC and did not seem to have influenced the choice to prescribe one of them [55]. A European cross-national drug utilization study reported [39] an increase in NOAC users (incidence from 8.7 per 10,000 in Spain to 27.6 per 10,000 in Denmark), with the highest increase observed for apixaban, followed by rivaroxaban. Overall, rivaroxaban presented the highest incidence, followed by apixaban and dabigatran. The authors correlate the differences detected among countries with different national or regional recommendations, prescription patterns and characteristics of the selected databases.

Interestingly, several studies reported that different factors affected the choice of NOACs in the observed population. The oldest patients were preferably treated with apixaban or edoxaban [56,57,58]. Moreover, in patients with comorbidities and higher stroke and bleeding risk, the drug of choice is apixaban, whereas those on rivaroxaban showed an intermediate clinical profile between apixaban and dabigatran [59]. These studies did not include edoxaban, the newest agent, with fewer available real-life data and a shorter follow-up duration. Comparative evaluations of NOACs have shown that, while dabigatran, rivaroxaban and apixaban provide similar efficacy in preventing stroke and systemic embolism in patients with atrial fibrillation, differences in their safety profiles are clinically relevant. In adjusted indirect comparisons of pivotal randomized trials, apixaban was associated with a significantly lower risk of major hemorrhage when compared with dabigatran and rivaroxaban [60]. Real-world studies further corroborate these findings, showing that bleeding rates, particularly gastrointestinal and intracranial events, vary across agents, even after careful propensity-score adjustment [61].

Data about Italian patients with NVAF who were treated with NOACs were collected into the AIFA database of monitoring registries and were published, including factors associated with treatment choice, treatment discontinuation and switches among drugs [59,62,63]. The observed population included very elderly patients at high risk of events, treated mostly with rivaroxaban (33.8% of treatments), followed by apixaban (31.1%), dabigatran (28.6%) and edoxaban (6.5%); the last was introduced on the market in 2016. Even this study showed that different factors affected the choice of a specific NOAC in the observed population, e.g., oldest patients were preferably treated with apixaban or edoxaban, in line with previous observations [56,57,58]; a CHA_2_DS_2_-VASc score equal to three or higher was more strongly associated with apixaban or dabigatran and a HAS-BLED score higher than four, with a higher frequency of prescription for apixaban and rivaroxaban. It is known that the occurrence of ADRs can be influenced by several factors: for example, being a frail elderly patient with multi-morbidity and exposure to polypharmacy [64,65]. Therefore, the different baseline characteristics that were demonstrated to influence the choice of NOACs could determine a different risk of reporting adverse events, as observed in this study. We have no data on concomitant medications or diseases, but we found a high prevalence of ADRs in elderly and very elderly subjects with all medications: in particular, with edoxaban and acenocumarol. This may be explained by the fact that OACs are commonly prescribed in older adults, due to their approved indications [66]. ADRs due to underlying diseases or patient characteristics, as well as those related to medication errors, drug–drug interactions, poor adherence and poor monitoring of patients, may be preventable [67]. An observational study recently conducted in five European countries [68] raised concerns about the level of adherence in clinical practice to restrictions, special warnings and precautions based on the medicines’ summary of product characteristics (SPCs), with a consequent possible increase in ADRs risk. Moreover, available ‘real world’ data suggest variable adherence to NOACs (from 38% to 99%) [69]. Strict adherence is crucial, as non-adherence to NOACs has been associated with increased risks of stroke, bleeding and death [70,71]. The poor adherence to NOACs can be explained by the absence of the need for routine coagulation monitoring [55,72]. This is one of the main advantages compared to VKAs, but can represent a risk for older and frail patients who require vigilance in order to balance the bleeding risk and anticoagulant effect and prevent potentially severe complications [69,73]. In this regard, the guidelines [69,74] recommend to regularly review patients treated with NOAC: in particular, after one month initially, and at least every three to four months thereafter. As clinical experience with NOACs grows, follow-up intervals may become longer, based on individual (patient-specific) or local (center-specific) factors.

Our analysis confirms the similarity of the safety profile of VKAs, significantly associated with the ADRs ‘Inv’ and ‘Inj&P’. This is an expected result, as a frequently reported ADR with VKA is related to indentification of international normalized ratios (INR) outside the therapeutic range [75]. Warfarin safety, indeed, is highly dependent on maintaining an optimal time in the therapeutic range (TTR), which can vary considerably across populations. This variability is influenced by numerous factors, including patient adherence, comorbidities, pharmacogenetic differences, concomitant medications, and, importantly, the healthcare system’s ability to provide adequate monitoring, education and support. Pharmacologic interactions also play a critical role. Therefore, differences in TTR may reflect not only individual patient characteristics but also the overall quality and structure of anticoagulation management programs across countries.

Moreover, our results suggest the similarity of the overall safety profile of dabigatran and rivaroxaban, which differs from that of the other NOACs, for the risk of reporting, above all, the ‘Gastr’ and ‘Nerv’ ADRs. Previously conflicting results have been reported [52,76,77,78,79,80,81]. Relative differences in bleeding sites among NOACs were found in previous studies [15,82,83,84,85,86]. Gastrointestinal bleeding remains a major concern of NOAC use, being potentially severe and even fatal [87,88]. At the same time, the possible non-adherence to NOACs due to gastrointestinal bleeding may increase the risk of stroke and VTE [89]. However, existing findings from meta-analyses of RCTs are heterogeneous [90].

Our findings are supported by the results published by EMA in 2019 about an observational study conducted on six databases covering regionally/nationally representative populations in five European countries, assessing the risk of major bleeding with the NOACs apixaban, dabigatran and rivaroxaban in patients with NVAF, in comparison with other OACs [68]. This study showed differences in the risk of major bleeding among these medicines. In particular, apixaban was not associated with an increased risk of gastrointestinal bleeding and seemed to be associated with the lowest risk of major events compared to dabigatran and rivaroxaban. Edoxaban was not included in the analysis. The separation of apixaban is guided by ‘Surg’ events. A lot of ADRs included in this cathegory are measures to manage ADRs (such as transfusions) or probably concomitant events (cardioversion or other cardiac procedures, dental care, surgery procedures not specified) without a clear causal/effect explaination. According to international guidelines, patient characteristics and surgical factors need to be taken into account to determine when to discontinue and restart a NOAC [69,74], in order to balance the risk of procedural bleeding with the risk for thromboembolism [90]. The PAUSE trial demonstrated that in patients treated with NOAC, a simple standardized perioperative management approach (without heparin bridging or coagulation function testing) is associated with low rates of bleeding and thromboembolism [91]. While invasive interventions require temporary discontinuation, less invasive procedures with a low bleeding risk do not necessarily require the suspension of treatment. In general, minor surgical procedures and those procedures where bleeding is easily controllable can be performed 12–24 h after the last NOAC intake, whereas in case of invasive procedures with a high risk of major bleeding, it is recommended to take the last NOAC dose 48 h or longer before surgery. After a procedure with immediate and complete haemostasis, NOACs can generally be restarted 6–8 h after the end of the intervention, with some exceptions. If an emergency intervention is required, the NOAC should be discontinued immediately. There are no specific indications to treat patients undergoing surgery with one NOAC or another. Therefore, the higher ADR-reporting in apixaban should be further investigated. Interestingly, we found differences in terms of fatal ADRs among drugs, with dabigatran associated with the highest risk of reporting an ADR with a fatal outcome. As expected, the most frequently reported fatal ‘Gastr’ and ‘Nerv’ ADRs were bleeding. Even in this case, the most important fatal ADR reported for apixaban (‘Genrl’) is not clearly explainable, being related to a generic ‘death’. This raises some concern about the quality of data and the need for further investigations in order to clarify if this event should have been classified as the outcome of another specific ADR, or should represent an ADR itself. We can observe a clear signal for Fatal ‘Psych’ ADRs and warfarin, almost all represented by ‘suicide’. Warfarin overdoses (intentional or unintentional) are relatively uncommon [92], but represent a major concern due to the potential significant morbidity and mortality. Emergency treatment is complicated by the risk of thromboembolic events, in the case of complete anticoagulation reversal [93]. Previous studies showed a high prevalence of depressive symptoms in patients treated with anticoagulants, with detrimental effects on anticoagulation therapy, due to all the above risks of poor adherence and consequent adverse medical events [94]. Screening for depression and appropriate treatment should be included in the management of these patients. Previous studies found non-bleeding adverse events associated with NOACs to be linked to multiple organ systems, including the reproductive system, endocrine disorders, and psychiatric disorders, which requires high vigilance in clinical practice [95].

Large variations have been found for fatal ‘Preg’ ADRs (in particular, ‘fetal death’ and ‘stillbirth’) and warfarin and acenocumarol use. Pregnant women frequently require anticoagulation during pregnancy or in postpartum, due to an increased risk of venous thromboembolism [96,97]. The use of anticoagulants during pregnancy is challenging; due to the potential teratogenic effects of drugs and the pharmacokinetic changes, there is a consequent need for complex dose adjustments [98]. VKAs are not considered to be acceptable in pregnancy [99], as they are able to cross the placenta and cause a typical gestation embryopathy, as well as other adverse fetal outcomes and bleeding [100]. Moreover, current guidelines advise against NOAC use too, even if their efficacy and safety profile during pregnancy is unknown, due to the exclusion of pregnant women from clinical trials [74,101,102], and low-molecular-weight heparin is still the treatment of choice during pregnancy [103].

Finally, the fewer rates of ADRs (and, in particular, of ‘Preg’ and ‘Hepatobiliary disorders’) might be responsible for the large deviation detected for edoxaban, and it is difficult to draw any conclusions. The differences observed in adverse event profiles suggest that OAC may have practical relevance for treatment individualization. In particular, the lower reporting rates of major gastrointestinal and central nervous system events with apixaban suggest a potentially safer option in elderly or frail patients, or in those with prior bleeding events. Conversely, the higher rates of fatal gastrointestinal ADRs associated with dabigatran and rivaroxaban highlight the importance of careful patient selection, avoidance of concomitant gastrotoxic agents, and regular reassessment of renal function. The distinct pattern of procedural and surgical-related events with apixaban further underlines the need for optimized perioperative management and clinician awareness of drug-specific risks. The present observations and the differences in ADRs’ sites found in this study help to understand the safety of anticoagulants in real life. Studies conducted on the spontaneous reporting system are affected by important limitations [104]. Interpretation of data found in the pharmacovigilance databases may be difficult due to a lot of biases, such as stimulated reporting, selective reporting and under-reporting [105]. Generally, only a small fraction of ADRs are reported [106], and the number of ADRs may be influenced by many different factors: first of all, the use of the product, the country-specific limitation of prescription, the frequency and the seriousness of reactions. Moreover, it is not always possible to assess the use of other suspected or concomitant medications that interact with the drug or contribute to the development of the ADR or underlying diseases, such as gastrointestinal tumors [107,108,109,110,111,112]. Neither clinical details of patients nor information on possible confounding factors and causality assessment are available. Furthermore, we found some other criticism for data interpretation: in particular, in the case of ADRs reported with apixaban. Despite these limitations, post-marketing surveillance still represents an important instrument to evaluate the real-world effectiveness and safety of drugs, especially in long-term use by unselected patients [106,113,114], as already demonstrated by our group in other settings [25,26,27,115,116,117,118]. To our knowledge, these are the first spontaneous reports related to all OACs identified in the EudraVigilance database. Future pharmacovigilance efforts should aim to integrate spontaneous reporting data with clinical and demographic information derived from electronic health records or dedicated registries. Such linkage would enable more robust causal inference, stratification by comorbidities, and assessment of confounding factors such as concomitant drug use, adherence and renal function. In addition, prospective real-world studies and active surveillance programs could help confirm the observed safety differences among individual oral anticoagulants and refine evidence-based prescribing recommendations.

Our study is informative and provides important data about overall and specific drug reporting in Europe. Indeed, EudraVigilance is one of the biggest spontaneous reporting systems in the world, with more than 14.5 million ICSRs collected till 2018 [119]. Our study is coherent with results from pre- and post-marketing studies, even though we identified some new potential safety signals and/or biases, for which further in-depth analysis should be performed. In the future, it would be useful to analyze nationwide data in order to investigate the difference in terms of ADR-reporting among European countries according to the possible variability in OACs use in Europe.

## 5. Conclusions

Our analysis of EudraVigilance data confirms that oral anticoagulants are associated with a high proportion of serious and fatal adverse drug reactions, with relevant differences among individual agents. VKAs showed a distinctive safety profile compared with NOACs, while within NOACs, dabigatran and rivaroxaban appeared more similar, and apixaban emerged as distinct, particularly regarding surgical and gastrointestinal events. These findings underline the importance of post-marketing surveillance to capture real-world safety signals that are not evident in randomized clinical trials. Further pharmacoepidemiological studies linking spontaneous reports with clinical data are warranted to refine the risk–benefit assessment and support safer therapeutic strategies.

## Figures and Tables

**Table 1:** Population characteristics as indicated in the individual case safety reports (ICSRs), according to drug. F: female; M: male; NS: not specified.

	Warfarin (%)	Acenocumarol (%)	Dabigatran (%)	Rivaroxaban (%)	Apixaban (%)	Edoxaban (%)	Total (%)
**ICSRs**	41,775 (17.1)	8818 (3.6)	48,341 (19.8)	101,577 (41.6)	39,741 (16.3)	3897 (1.6)	244,149 (100)
**Gender**							
**F**	19,745 (47.3)	4194 (47.6)	21,284 (44.0)	45,438 (44.7)	18,852 (47.4)	1870 (48.0)	111,383 (45.6)
**M**	19,789 (47.4)	4449 (50.5)	23,595 (48.8)	45,952 (45.2)	18,692 (47.0)	1936 (49.7)	114,413 (46.9)
**NS**	2241 (5.4)	175 (2.0)	3462 (7.2)	10,187 (10.0)	2197 (5.5)	91 (2.3)	183,53 (7.5)
**Age**							
**Over 85**	7132 (17.1)	1708 (19.4)	7854 (16.2)	10,819 (10.7)	7369 (18.5)	760 (19.5)	35,642 (14.6)
**65–85**	21,240 (50.8)	5049 (57.3)	24,389 (50.5)	45,197 (44.5)	18,634 (46.9)	2040 (52.3)	116,549 (47.7)
**18–64**	8452 (20.2)	1580 (17.9)	5107 (10.6)	20,184 (19.9)	4501 (11.3)	415 (10.6)	40,239 (16.5)
**<18**	325 (0.8)	46 (0.5)	26 (0.1)	108 (0.1)	26 (0.1)	5 (0.1)	536 (0.2)
**NS**	4626 (11.1)	435 (4.9)	10,965 (22.7)	25,269 (24.9)	9211 (23.2)	677 (17.4)	51,183 (21.0)

**Table 2:** Seriousness and outcome of suspected adverse drug reactions (ADRs) associated with oral anticoagulants. ADR: adverse drug reactions; NS: not specified.

	Warfarin (%)	Acenocumarol (%)	Dabigatran (%)	Rivaroxaban (%)	Apixaban (%)	Edoxaban (%)	Total (%)
**ADRs**	81,100 (18.8)	17,294 (4.0)	87,140 (20.2)	172,624 (40.0)	67,104 (15.6)	6092 (1.4)	431,354 (100.0)
**Seriousness**							
**Serious**	73,784 (91.0)	15,357 (88.8)	81,660 (93.7)	164,925 (95.5)	60,991 (90.9)	4322 (70.9)	401,039 (93.0)
**Non-serious**	7232 (8.9)	1918 (11.1)	5475 (6.3)	7699 (4.5)	6113 (9.1)	1770 (29.1)	30,207 (7)
**NS**	84 (0.1)	19 (0.1)	5 (0.0)	0 (0.0)	0 (0.0)	0 (0.0)	108 (0.0)
**Outcome**							
**Fatal**	6817 (8.4)	1466 (8.5)	10,774 (12.4)	12,947 (6.6)	5878 (8.8)	368 (6.0)	38,250 (8.9)
**Not recovered**	5747 (7.3)	969 (5.8)	10,882 (13.4)	13,382 (8.0)	5129 (8.1)	841 (14.3)	36,950 (9.0)
**Not specified**	2164 (2.8)	584 (3.5)	12 (0.0)	221 (0.1)	0 (0.0)	0 (0.0)	2981 (0.7)
**Recovered**	22,603 (28.8)	8123 (48.9)	25,083 (30.9)	35,182 (21.1)	10,005 (15.8)	1728 (29.3)	102,724 (24.9)
**Recovered with sequelae**	1014 (1.3)	410 (2.5)	1561 (1.9)	2085 (1.2)	945 (1.5)	180 (3.1)	6195 (1.5)
**Recovering**	12,250 (15.6)	2231 (13.4)	3118 (3.8)	26,037 (15.6)	5337 (8.4)	858 (14.6)	49,831 (12.1)
**Unknown**	34,752 (44.3)	4301 (25.9)	40,472 (49.9)	89,991 (53.9)	42,020 (66.2)	2284 (38.8)	213,820 (51.8)

**Table 3:** Percentage of individual case safety reports (ICSRs), according to European country and drug.

Drug	France	Italy	Germany	UK
**Warfarin**	19.8%	**57.2%**	0.7%	18.8%
**Acenocumarol**	11.6%	7.2%	0.2%	0.2%
**Dabigatran**	15.6%	15.8%	16.6%	13.3%
**Rivaroxaban**	**37.7%**	10%	49.1%	40.4%
**Apixaban**	15.2%	7.6%	28.3%	24.7%
**Edoxaban**	0%	2.1%	5.1%	2.6%
**Total**	100%	100%	100%	100%

**Table 4:** Percentages of indexed residuals for ADRs, categorized according to system organ classes (SOCs).

SOC	Warfarin	Acenocumarol	Dabigatran	Rivaroxaban	Apixaban	Edoxaban
**Blood**	17.3%	86.6%	28.3%	−14.8%	−41.9%	0.2%
**Card**	−25.1%	−35.9%	54.7%	−22.9%	30.0%	−28.2%
**Cong**	27.7%	−49.5%	−37.9%	40.9%	−70.2%	−72.7%
**Ear**	−13.6%	−36.6%	−27.6%	2.2%	51.2%	54.9%
**Endo**	52.3%	−12.0%	−33.8%	−7.6%	5.2%	−21.1%
**Eye**	−3.1%	−19.8%	−31.9%	5.9%	34.9%	1.9%
**Gastr**	−32.3%	−20.0%	27.1%	15.2%	−29.6%	−6.0%
**Genrl**	20.5%	24.1%	−14.1%	−11.0%	17.3%	−18.2%
**Hepato**	−9.0%	8.3%	−1.1%	−1.0%	6.4%	69.1%
**Immun**	17.7%	−31.4%	−30.4%	−7.1%	37.2%	80.9%
**Infec**	−9.6%	−46.2%	47.0%	−20.0%	14.8%	−8.9%
**Inj&P**	4.4%	−10.4%	−18.0%	−1.0%	23.9%	−7.3%
**Inv**	104.0%	128.0%	−35.0%	−28.5%	−38.8%	−11.9%
**Metab**	36.3%	5.1%	32.1%	−28.8%	−10.3%	−27.0%
**Musc**	7.2%	−0.1%	−35.1%	7.6%	17.0%	3.4%
**Neopl**	−39.9%	−62.1%	56.2%	−24.1%	54.3%	−10.8%
**Nerv**	−18.6%	−28.0%	8.3%	−4.4%	26.3%	43.0%
**Preg**	255.4%	149.8%	−94.5%	−59.9%	−64.2%	−68.5%
**Product**	40.2%	−39.7%	−9.7%	−0.3%	−17.6%	−79.8%
**Psych**	24.3%	8.0%	−12.8%	−14.4%	19.8%	27.2%
**Renal**	−18.1%	−8.4%	11.2%	14.5%	−27.3%	−5.2%
**Repro**	−36.2%	−56.7%	−58.2%	67.6%	−38.1%	−21.4%
**Resp**	7.3%	−17.4%	−16.3%	11.2%	−12.4%	6.7%
**Skin**	0.1%	11.9%	−28.3%	−3.1%	30.4%	122.6%
**Surg**	−32.3%	−45.6%	−41.7%	−17.8%	156.1%	−56.2%
**Vasc**	−8.4%	−19.9%	−12.0%	13.8%	−1.1%	−37.1%

Blood: blood and lymphatic system disorders; Card: cardiac disorders; Cong: congenital, familial and genetic disorders; Ear: ear and labyrinth disorders; Endo: endocrine disorders; Eye: eye disorders; Gastr: gastrointestinal disorders; Genrl: general disorders and administration site conditions; Hepato: hepatobiliary disorders; Immun: immune system disorders; Infec: infections and infestations; Inj&P: injury, poisoning and procedural complications; Inv: investigations; Metab: metabolism and nutrition disorders; Musc: musculoskeletal and connective tissue disorders; Neopl: neoplasms benign, malignant and unspecified (incl cysts and polyps); Nerv: nervous system disorders; Preg: pregnancy, puerperium and perinatal conditions; Product: product issues; Psych: psychiatric disorders; Renal: renal and urinary disorders; Repro: reproductive system and breast disorders; Resp: respiratory, thoracic and mediastinal disorders; Skin: skin and subcutaneous tissue disorders; Surg: surgical and medical procedures; Vasc: vascular disorders.

**Table 5:** Percentages of indexed residuals for fatal ADRs categorized according to system organ classes (SOCs).

SOC	Warfarin	Acenocumarol	Dabigatran	Rivaroxaban	Apixaban	Edoxaban
**Blood**	6.4%	77.9%	44.8%	−24.9%	−52.3%	8.2%
**Card**	−21.0%	−25.7%	34.2%	−10.0%	−8.8%	−36.3%
**Cong**	209.7%	53.5%	−52.4%	−28.6%	−82.7%	−100.0%
**Endo**	106.2%	127.2%	−85.9%	29.1%	−48.7%	−100.0%
**Gastr**	−32.4%	−15.0%	53.9%	−2.7%	−50.5%	−30.8%
**Genrl**	−19.2%	−43.4%	−13.7%	−16.7%	95.3%	−38.3%
**Hepato**	−14.1%	62.3%	−3.1%	2.7%	−10.7%	191.1%
**Immun**	167.3%	89.3%	−6.0%	−60.9%	−57.2%	−100.0%
**Infec**	−27.3%	−34.7%	39.2%	−7.4%	−18.1%	21.2%
**Inj&P**	69.1%	49.1%	−25.5%	−4.7%	−31.6%	−3.1%
**Metab**	43.2%	70.0%	26.6%	−33.9%	−32.3%	−100.0%
**Musc**	74.8%	309.1%	−64.1%	4.4%	−40.2%	−100.0%
**Neopl**	−29.1%	−75.2%	7.1%	−14.6%	66.8%	27.4%
**Nerv**	19.4%	20.8%	−36.9%	19.3%	−4.1%	40.5%
**Preg**	386.1%	72.1%	−89.3%	−91.1%	−100.0%	208.8%
**Psych**	292.4%	25.7%	−82.6%	−45.8%	−76.6%	−100.0%
**Renal**	−31.1%	37.2%	48.1%	−6.4%	−45.2%	−31.3%
**Repro**	−9.1%	35.2%	45.5%	−6.9%	−59.3%	−100.0%
**Resp**	−26.1%	−22.6%	2.1%	21.7%	−20.9%	63.2%
**Skin**	135.1%	97.0%	−24.6%	−40.6%	−44.4%	76.7%
**Vasc**	−21.6%	12.0%	27.9%	1.1%	−30.3%	−20.9%

Blood: blood and lymphatic system disorders; Card: cardiac disorders; Cong: congenital, familial and genetic disorders; Endo: endocrine disorders; Gastr: gastrointestinal disorders; Genrl: general disorders and administration site conditions; Hepato: hepatobiliary disorders; Immun: immune system disorders; Infec: infections and infestations; Inj&P: injury, poisoning and procedural complications; Inv: investigations; Metab: metabolism and nutrition disorders; Musc: musculoskeletal and connective tissue disorders; Neopl: neoplasms benign, malignant and unspecified (incl cysts and polyps); Nerv: nervous system disorders; Preg: pregnancy, puerperium and perinatal conditions; Psych: psychiatric disorders; Renal: renal and urinary disorders; Repro: reproductive system and breast disorders; Resp: respiratory, thoracic and mediastinal disorders; Skin: skin and subcutaneous tissue disorders; Vasc: vascular disorders.

## Data Availability

The data that support the findings of this study are available at http://www.adrreports.eu/en/search.html (accessed on 14 October 2025). These data were derived from EudraVigilance, the European database of suspected adverse drug reaction reports, published by European Medicines Agency (EMA).

## Supplementary Materials

The following supporting information can be downloaded at: https://www.mdpi.com/article/10.3390/hematolrep17050054/s1, Table S1: Frequencies of individual ADRs, categorized according to system organ classes (SOCs), with percentages in brackets. Table S2: Frequencies of individual fatal ADRs, categorized according to system organ classes (SOCs), with percentages in brackets. Table S3: Rrisk of reporting (ROR) and theirs 95% confidence intervals (LCL, lower control limit, and UCL, upper control limit) for ADRs. ADRs reported into EudraVigilance for each drug were categorized according to system organ classes (SOCs), and have been compared with the related categories reported for the others. Table S4: Rrisk of reporting (ROR) and theirs 95% confidence intervals (LCL, lower control limit, and UCL, upper control limit) for fatal ADRs. ADRs reported into EudraVigilance for each drug were categorized according to system organ classes (SOCs), and have been compared with the related categories reported for the others. Figure S1: Contribution biplot without ‘Inv’.

## Abbreviations

The following abbreviations are used in this manuscript:

ADR	Adverse Drug Reaction
AF	Atrial Fibrillation
CA	Correspondence Analysis
CI	Confidence Interval
EEA	European Economic Area
EMA	European Medicines Agency
ICSR	Individual Case Safety Report
MedDRA	Medical Dictionary for Regulatory Activities
NOAC	Novel Oral Anticoagulant
OAC	Oral Anticoagulant
OR	Odds Ratio
ROR	Reporting Odds Ratio
SOC	System Organ Class
VKA	Vitamin K Antagonist
