# Supplementary material for: Vitamin K Antagonists (VKAs) and Novel Oral Anticoagulants (NOACs) Safety Comparison Based on Data from EudraVigilance Database
Source: Hematol Rep. 2025 Oct 18;17(5):54. doi: 10.3390/hematolrep17050054 (PMC12563034; doi:10.3390/hematolrep17050054)
Supplement: Supplementary file 1 [file hematolrep-17-00054-s001.zip › hematolrep-3901138-supplementary.pdf]

Table S1. Frequencies of individual ADRs. categorized according to system organ classes (SOCs). with percentages in brackets.

SOC	Warfarin	Acenocumarol	Dabigatran	Rivaroxaban	Apixaban	Edoxaban
**Blood**	4674 (5.8)	1587 (9.2)	5495 (6.3)	7225 (4.2)	1914 (2.9)	300 (4.9)
**Card**	2135 (2.6)	390 (2.3)	4743 (5.4)	4684 (2.7)	3067 (4.6)	154 (2.5)
**Cong**	249 (0.3)	21 (0.1)	130 (0.1)	585 (0.3)	48 (0.1)	4 (0.1)
**Ear**	230 (0.3)	36 (0.2)	207 (0.2)	579 (0.3)	333 (0.5)	31 (0.5)
**Endo**	154 (0.2)	19 (0.1)	72 (0.1)	199 (0.1)	88 (0.1)	6 (0.1)
**Eye**	1024 (1.3)	181 (1)	774 (0.9)	2383 (1.4)	1180 (1.8)	81 (1.3)
**Gastr**	9845 (12.2)	2484 (14.4)	19,857 (22.8)	35,673 (20.7)	8471 (12.6)	1028 (16.9)
**Genrl**	7800 (9.6)	1715 (9.9)	5979 (6.9)	12,268 (7.1)	6281 (9.4)	398 (6.5)
**Hepato**	594 (0.7)	151 (0.9)	694 (0.8)	1377 (0.8)	575 (0.9)	83 (1.4)
**Immun**	225 (0.3)	28 (0.2)	143 (0.2)	378 (0.2)	217 (0.3)	26 (0.4)
**Infec**	1465 (1.8)	186 (1.1)	2560 (2.9)	2761 (1.6)	1540 (2.3)	111 (1.8)
**Inj&P**	7025 (8.7)	1287 (7.4)	5934 (6.8)	14,192 (8.2)	6902 (10.3)	469 (7.7)
**Inv**	12,108 (14.9)	2889 (16.7)	4146 (4.8)	9031 (5.2)	3005 (4.5)	393 (6.5)
**Metab**	1142 (1.4)	188 (1.1)	1190 (1.4)	1270 (0.7)	622 (0.9)	46 (0.8)
**Musc**	2056 (2.5)	409 (2.4)	1337 (1.5)	4392 (2.5)	1855 (2.8)	149 (2.4)
**Neopl**	475 (0.6)	64 (0.4)	1327 (1.5)	1277 (0.7)	1009 (1.5)	53 (0.9)
**Nerv**	8167 (10.1)	1542 (8.9)	11,673 (13.4)	20406 (11.8)	10,474 (15.6)	1078 (17.7)
**Preg**	300 (0.4)	45 (0.3)	5 (0.01)	72 (0.04)	25 (0.04)	2 (0.03)
**Product**	185 (0.2)	17 (0.1)	128 (0.1)	280 (0.2)	90 (0.1)	2 (0.03)
**Psych**	1105 (1.4)	205 (1.2)	833 (1)	1620 (0.9)	881 (1.3)	85 (1.4)
**Renal**	2964 (3.7)	708 (4.1)	4327 (5)	8826 (5.1)	2179 (3.3)	258 (4.2)
**Repro**	670 (0.8)	97 (0.6)	472 (0.5)	3746 (2.2)	538 (0.8)	62 (1)
**Resp**	6089 (7.5)	1000 (5.8)	5102 (5.9)	13,429 (7.8)	4110 (6.1)	455 (7.5)
**Skin**	2464 (3)	588 (3.4)	1898 (2.2)	5077 (2.9)	2656 (4)	412 (6.8)
**Surg**	862 (1.1)	148 (0.9)	798 (0.9)	2229 (1.3)	2699 (4)	42 (0.7)
**Vasc**	6991 (8.6)	1305 (7.5)	7217 (8.3)	18,493 (10.7)	6247 (9.3)	361 (5.9)
**Total**	80,998 (100)	17,290 (100)	87,041 (100)	172,452 (100)	67,006 (100)	6089 (100)

Blood = Blood and lymphatic system disorders; Card = Cardiac disorders; Cong = Congenital. familial and genetic disorders; Ear = Ear and labyrinth disorders; Endo = Endocrine disorders; Eye = Eye disorders; Gastr = Gastrointestinal disorders; Genrl = General disorders and administration site conditions; Hepato = Hepatobiliary disorders; Immun = Immune system disorders; Infec = Infections and infestations; Inj&P = Injury. poisoning and procedural complications; Inv = Investigations; Metab = Metabolism and nutrition disorders; Musc = Musculoskeletal and connective tissue disorders; Neopl = Neoplasms benign. malignant and unspecified (incl cysts and polyps); Nerv = Nervous system disorders; Preg = Pregnancy. puerperium and perinatal conditions; Product = Product issues; Psych = Psychiatric disorders; Renal = Renal and urinary disorders; Repro = Reproductive system and breast disorders; Resp = Respiratory. thoracic and mediastinal disorders; Skin = Skin and subcutaneous tissue disorders; Surg = Surgical and medical procedures; Vasc = Vascular disorders.

Table S2. Frequencies of individual fatal ADRs. categorized according to system organ classes (SOCs) . with percentages in brackets.

SOC	Warfarin	Acenocumarol	Dabigatran	Rivaroxaban	Apixaban	Edoxaban
**Blood**	175 (2.7)	59 (4.5)	387 (3.6)	241 (1.9)	70 (1.2)	10 (2.7)
**Card**	353 (5.4)	67 (5.1)	975 (9.2)	786 (6.2)	364 (6.2)	16 (4.4)
**Cong**	20 (0.3)	2 (0.2)	5 (0.05)	9 (0.1)	1 (0.02)	0 (0)
**Endo**	9 (0.1)	2 (0.2)	1 (0.01)	11 (0.1)	2 (0.03)	0 (0)
**Gastr**	556 (8.5)	141 (10.7)	2057 (19.4)	1563 (12.3)	364 (6.2)	32 (8.7)
**Genrl**	955 (14.6)	135 (10.3)	1658 (15.6)	1923 (15.1)	2063 (35.4)	41 (11.2)
**Hepato**	42 (0.6)	16 (1.2)	77 (0.7)	98 (0.8)	39 (0.7)	8 (2.2)
**Immun**	7 (0.1)	1 (0.1)	4 (0.04)	2 (0.02)	1 (0.02)	0 (0)
**Infec**	160 (2.5)	29 (2.2)	498 (4.7)	398 (3.1)	161 (2.8)	15 (4.1)
**Inj&P**	714 (10.9)	127 (9.6)	511 (4.8)	786 (6.2)	258 (4.4)	23 (6.3)
**Metab**	71 (1.1)	17 (1.3)	102 (1)	64 (0.5)	30 (0.5)	0 (0)
**Musc**	36 (0.6)	17 (1.3)	12 (0.1)	42 (0.3)	11 (0.2)	0 (0)
**Neopl**	99 (1.5)	7 (0.5)	243 (2.3)	233 (1.8)	208 (3.6)	10 (2.7)
**Nerv**	2131 (32.6)	435 (33)	1830 (17.3)	4157 (32.6)	1529 (26.2)	141 (38.4)
**Preg**	28 (0.4)	2 (0.2)	1 (0.01)	1 (0.01)	0 (0)	1 (0.3)
**Psych**	263 (4)	17 (1.3)	19 (0.2)	71 (0.6)	14 (0.2)	0 (0)
**Renal**	107 (1.6)	43 (3.3)	374 (3.5)	284 (2.2)	76 (1.3)	6 (1.6)
**Repro**	10 (0.2)	3 (0.2)	26 (0.2)	20 (0.2)	4 (0.1)	0 (0)
**Resp**	298 (4.6)	63 (4.8)	669 (6.3)	958 (7.5)	285 (4.9)	37 (10.1)
**Skin**	71 (1.1)	12 (0.9)	37 (0.3)	35 (0.3)	15 (0.3)	3 (0.8)
**Vasc**	423 (6.5)	122 (9.3)	1122 (10.6)	1065 (8.4)	336 (5.8)	24 (6.5)
**Total**	6528 (100)	1317 (100)	10,608 (100)	12,747 (100)	5831 (100)	367 (100)

Blood = Blood and lymphatic system disorders; Card = Cardiac disorders; Cong = Congenital. familial and genetic disorders; Endo = Endocrine disorders; Gastr = Gastrointestinal disorders; Genrl = General disorders and administration site conditions; Hepato = Hepatobiliary disorders; Immun = Immune system disorders; Infec = Infections and infestations; Inj&P = Injury. poisoning and procedural complications; Inv = Investigations; Metab = Metabolism and nutrition disorders; Musc = Musculoskeletal and connective tissue disorders; Neopl = Neoplasms benign. malignant and unspecified (incl cysts and polyps); Nerv = Nervous system disorders; Preg = Pregnancy. puerperium and perinatal conditions; Psych = Psychiatric disorders; Renal = Renal and urinary disorders; Repro = Reproductive system and breast disorders; Resp = Respiratory. thoracic and mediastinal disorders; Skin = Skin and subcutaneous tissue disorders; Vasc = Vascular disorders.

Table S3. Rrisk of reporting (ROR) and theirs 95% confidence intervals (LCL. lower control limit. and UCL. upper control limit) for ADRs. ADRs reported into EudraVigilance for each drug were categorized according to system organ classes (SOCs). and have been compared with the related categories reported for the others.

	Warfarin			Acenocumarol			Dabigatran			Rivaroxaban			Apixaban			Edoxaban		
**ADR**	**ROR**	**LCL**	**UCL**	**ROR**	**LCL**	**UCL**	**ROR**	**LCL**	**UCL**	**ROR**	**LCL**	**UCL**	**ROR**	**LCL**	**UCL**	**ROR**	**LCL**	**UCL**
**Blood**	1.24	1.20	1.28	2.03	1.92	2.14	1.43	1.38	1.48	0.76	0.74	0.78	0.53	0.50	0.56	0.98	0.87	1.11
**Card**	0.70	0.66	0.73	0.61	0.55	0.69	1.81	1.74	1.88	0.65	0.62	0.67	1.50	1.44	1.57	0.74	0.63	0.88
**Cong**	1.27	1.10	1.48	0.46	0.29	0.72	0.59	0.49	0.71	1.99	1.76	2.26	0.27	0.20	0.36	0.27	0.10	0.73
**Ear**	0.83	0.72	0.96	0.62	0.44	0.86	0.71	0.61	0.82	1.01	0.91	1.13	1.67	1.48	1.89	1.52	1.06	2.17
**Endo**	1.69	1.39	2.05	0.81	0.50	1.31	0.67	0.52	0.86	0.85	0.71	1.01	1.09	0.87	1.38	0.80	0.36	1.79
**Eye**	0.95	0.89	1.02	0.78	0.68	0.91	0.66	0.61	0.71	1.08	1.02	1.14	1.45	1.36	1.55	0.99	0.79	1.24
**Gastr**	0.58	0.56	0.59	0.75	0.72	0.79	1.45	1.42	1.48	1.38	1.35	1.40	0.63	0.62	0.65	0.93	0.87	1.00
**Genrl**	1.45	1.41	1.49	1.48	1.40	1.56	0.75	0.73	0.78	0.87	0.84	0.89	0.98	0.94	1.01	0.88	0.79	0.98
**Hepato**	0.89	0.82	0.98	1.04	0.88	1.24	0.99	0.91	1.08	0.98	0.91	1.05	1.10	1.00	1.20	1.64	1.30	2.06
**Immun**	1.19	1.03	1.38	0.65	0.45	0.96	0.67	0.56	0.80	0.88	0.77	1.00	1.49	1.29	1.74	1.81	1.22	2.67
**Infec**	0.92	0.87	0.98	0.51	0.44	0.60	1.63	1.55	1.72	0.69	0.65	0.72	1.26	1.19	1.33	0.89	0.73	1.09
**Inj&P**	1.00	0.97	1.03	0.84	0.79	0.89	0.79	0.77	0.81	0.98	0.96	1.00	1.39	1.35	1.43	0.91	0.83	1.00
**Inv**	2.99	2.92	3.06	2.69	2.57	2.80	0.61	0.59	0.63	0.56	0.55	0.58	0.55	0.53	0.57	0.84	0.76	0.94
**Metab**	1.49	1.39	1.59	1.01	0.87	1.18	1.47	1.37	1.57	0.59	0.55	0.63	0.90	0.82	0.98	0.75	0.56	1.01
**Musc**	1.08	1.02	1.13	0.96	0.86	1.06	0.62	0.59	0.66	1.11	1.07	1.16	1.22	1.16	1.29	1.02	0.86	1.20
**Neopl**	0.53	0.48	0.59	0.40	0.31	0.52	1.95	1.81	2.10	0.64	0.60	0.69	1.68	1.55	1.82	0.87	0.64	1.17
**Nerv**	0.67	0.65	0.69	0.59	0.56	0.63	1.26	1.23	1.29	0.87	0.85	0.89	1.49	1.46	1.53	1.60	1.49	1.72
**Preg**	8.12	6.63	9.94	2.73	1.99	3.74	0.04	0.02	0.11	0.30	0.23	0.39	0.35	0.23	0.52	0.16	0.02	1.16
**Product**	1.53	1.30	1.82	0.59	0.36	0.95	0.92	0.76	1.12	0.97	0.83	1.13	0.80	0.64	1.00	0.19	0.05	0.77
**Psych**	1.03	0.95	1.11	1.07	0.92	1.24	0.96	0.89	1.03	0.81	0.76	0.86	1.36	1.26	1.46	1.36	1.09	1.69
**Renal**	0.78	0.75	0.81	0.88	0.82	0.96	1.14	1.10	1.19	1.29	1.25	1.33	0.69	0.66	0.72	0.94	0.83	1.07
**Repro**	0.58	0.53	0.63	0.41	0.33	0.50	0.36	0.33	0.40	3.09	2.92	3.27	0.58	0.53	0.63	0.77	0.60	0.99
**Resp**	1.13	1.10	1.17	0.81	0.76	0.87	0.77	0.75	0.80	1.20	1.17	1.23	0.85	0.83	0.89	1.03	0.94	1.14
**Skin**	0.97	0.93	1.02	1.11	1.02	1.20	0.69	0.66	0.73	0.93	0.90	0.97	1.41	1.35	1.48	2.31	2.08	2.56
**Surg**	0.62	0.58	0.67	0.52	0.44	0.62	0.55	0.51	0.59	0.71	0.68	0.75	3.71	3.53	3.90	0.42	0.31	0.57
**Vasc**	0.90	0.88	0.93	0.75	0.71	0.80	0.79	0.77	0.81	1.30	1.28	1.33	1.02	0.99	1.05	0.59	0.53	0.66

Blood = Blood and lymphatic system disorders; Card = Cardiac disorders; Cong = Congenital. familial and genetic disorders; Ear = Ear and labyrinth disorders; Endo = Endocrine disorders; Eye = Eye disorders; Gastr = Gastrointestinal disorders; Genrl = General disorders and administration site conditions; Hepato = Hepatobiliary disorders; Immun = Immune system disorders; Infec = Infections and infestations; Inj&P = Injury. poisoning and procedural complications; Inv = Investigations; Metab = Metabolism and nutrition disorders; Musc = Musculoskeletal and connective tissue disorders; Neopl = Neoplasms benign. malignant and unspecified (incl cysts and polyps); Nerv = Nervous system disorders; Preg = Pregnancy. puerperium and perinatal conditions; Product = Product issues; Psych = Psychiatric disorders; Renal = Renal and urinary disorders; Repro = Reproductive system and breast disorders; Resp = Respiratory. thoracic and mediastinal disorders; Skin = Skin and subcutaneous tissue disorders; Surg = Surgical and medical procedures; Vasc = Vascular disorders.

Table S4. Rrisk of reporting (ROR) and theirs 95% confidence intervals (LCL. lower control limit. and UCL. upper control limit) for fatal ADRs. ADRs reported into EudraVigilance for each drug were categorized according to system organ classes (SOCs). and have been compared with the related categories reported for the others.

	Warfarin			Acenocumarol			Dabigatran			Rivaroxaban			Apixaban			Edoxaban		
**ADR**	**ROR**	**LCL**	**UCL**	**ROR**	**LCL**	**UCL**	**ROR**	**LCL**	**UCL**	**ROR**	**LCL**	**UCL**	**ROR**	**LCL**	**UCL**	**ROR**	**LCL**	**UCL**
**Blood**	1.08	0.92	1.28	1.87	1.43	2.45	1.79	1.57	2.04	0.66	0.57	0.76	0.43	0.33	0.55	1.08	0.58	2.04
**Card**	0.74	0.66	0.83	0.72	0.56	0.93	1.61	1.48	1.75	0.85	0.78	0.92	0.89	0.79	1.00	0.62	0.37	1.02
**Cong**	5.58	2.92	10.65	1.57	0.38	6.52	0.39	0.15	1.01	0.62	0.29	1.32	0.15	0.02	1.10	0.00	0.00	0.00
**Endo**	2.66	1.18	6.03	2.38	0.56	10.1	0.11	0.01	0.78	1.52	0.69	3.35	0.47	0.11	2.00	0.00	0.00	0.00
**Gastr**	0.60	0.55	0.66	0.83	0.69	0.99	2.19	2.05	2.33	0.95	0.89	1.02	0.42	0.37	0.47	0.66	0.46	0.95
**Genrl**	0.74	0.68	0.79	0.51	0.42	0.61	0.78	0.74	0.83	0.72	0.68	0.77	3.12	2.93	3.32	0.57	0.41	0.78
**Hepato**	0.83	0.60	1.16	1.67	1.00	2.77	0.96	0.74	1.25	1.04	0.81	1.33	0.88	0.62	1.23	3.01	1.48	6.13
**Immun**	4.14	1.50	11.4	1.96	0.26	14.9	0.92	0.29	2.88	0.30	0.07	1.32	0.39	0.05	2.94	0.00	0.00	0.00
**Infec**	0.68	0.57	0.80	0.64	0.44	0.92	1.68	1.50	1.89	0.89	0.79	1.00	0.79	0.66	0.93	1.22	0.73	2.06
**Inj&P**	2.10	1.92	2.30	1.57	1.30	1.90	0.66	0.60	0.73	0.93	0.85	1.01	0.63	0.55	0.72	0.97	0.63	1.48
**Metab**	1.58	1.21	2.07	1.75	1.07	2.87	1.42	1.11	1.81	0.56	0.42	0.74	0.64	0.44	0.93	0.00	0.00	0.00
**Musc**	2.08	1.41	3.08	4.66	2.78	7.81	0.29	0.16	0.52	1.07	0.73	1.56	0.56	0.30	1.03	0.00	0.00	0.00
**Neopl**	0.66	0.54	0.82	0.24	0.11	0.50	1.10	0.95	1.29	0.79	0.68	0.92	1.94	1.65	2.27	1.29	0.68	2.42
**Nerv**	1.36	1.29	1.45	1.32	1.18	1.49	0.46	0.43	0.48	1.48	1.41	1.55	0.94	0.88	1.00	1.67	1.35	2.06
**Preg**	26.6	10.3	68.9	1.77	0.42	7.40	0.08	0.01	0.58	0.06	0.01	0.44	0.00	0.00	0.00	3.16	0.43	23.2
**Psych**	10.67	8.59	13.25	1.27	0.78	2.08	0.13	0.08	0.21	0.44	0.34	0.56	0.20	0.12	0.35	0.00	0.00	0.00
**Renal**	0.64	0.52	0.79	1.40	1.03	1.92	1.86	1.63	2.13	0.90	0.78	1.04	0.50	0.39	0.63	0.68	0.30	1.53
**Repro**	0.89	0.45	1.75	1.37	0.43	4.38	1.78	1.08	2.94	0.90	0.53	1.53	0.37	0.13	1.01	0.00	0.00	0.00
**Resp**	0.69	0.61	0.78	0.76	0.59	0.98	1.03	0.94	1.13	1.40	1.29	1.53	0.75	0.66	0.85	1.71	1.22	2.41
**Skin**	3.32	2.45	4.50	2.05	1.14	3.70	0.69	0.48	0.99	0.49	0.34	0.71	0.51	0.30	0.87	1.79	0.57	5.62
**Vasc**	0.73	0.66	0.81	1.14	0.94	1.38	1.49	1.38	1.61	1.02	0.94	1.10	0.64	0.57	0.72	0.77	0.51	1.17

Blood = Blood and lymphatic system disorders; Card = Cardiac disorders; Cong = Congenital. familial and genetic disorders; Endo = Endocrine disorders; Gastr = Gastrointestinal disorders; Genrl = General disorders and administration site conditions; Hepato = Hepatobiliary disorders; Immun = Immune system disorders; Infec = Infections and infestations; Inj&P = Injury. poisoning and procedural complications; Inv = Investigations; Metab = Metabolism and nutrition disorders; Musc = Musculoskeletal and connective tissue disorders; Neopl = Neoplasms benign. malignant and unspecified (incl cysts and polyps); Nerv = Nervous system disorders; Preg = Pregnancy. puerperium and perinatal conditions; Psych = Psychiatric disorders; Renal = Renal and urinary disorders; Repro = Reproductive system and breast disorders; Resp = Respiratory. thoracic and mediastinal disorders; Skin = Skin and subcutaneous tissue disorders; Vasc = Vascular disorders.

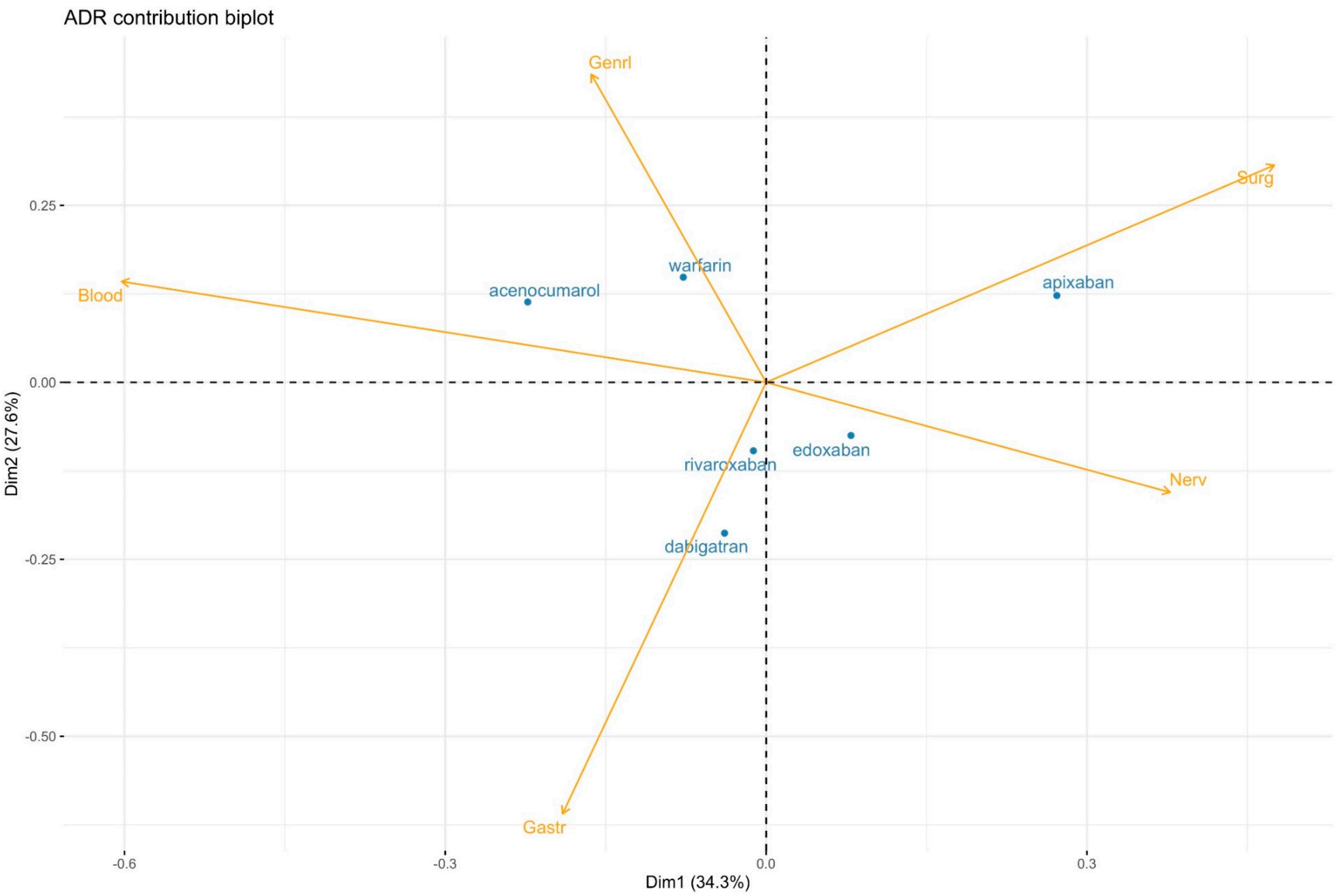

Figure S1. Contribution biplot without 'Inv'.
